# Sarcoidosis with multiple organ involvement

**DOI:** 10.1002/ccr3.1601

**Published:** 2018-05-28

**Authors:** Yve Mendes Rodrigues, Gláucia Zanetti, Edson Marchiori

**Affiliations:** ^1^ Universidade Federal do Rio de Janeiro Rio de Janeiro Brasil

**Keywords:** computed tomography, cutaneous disease, hepatosplenomegaly, pulmonary disease, sarcoidosis

## Abstract

This is an interesting case that highlights the variability of the clinical course of sarcoidosis and the importance of the knowledge of the clinical and radiologic features of the disease for its diagnosis and management.

## 1 CASE REPORT

**Figure 1:**
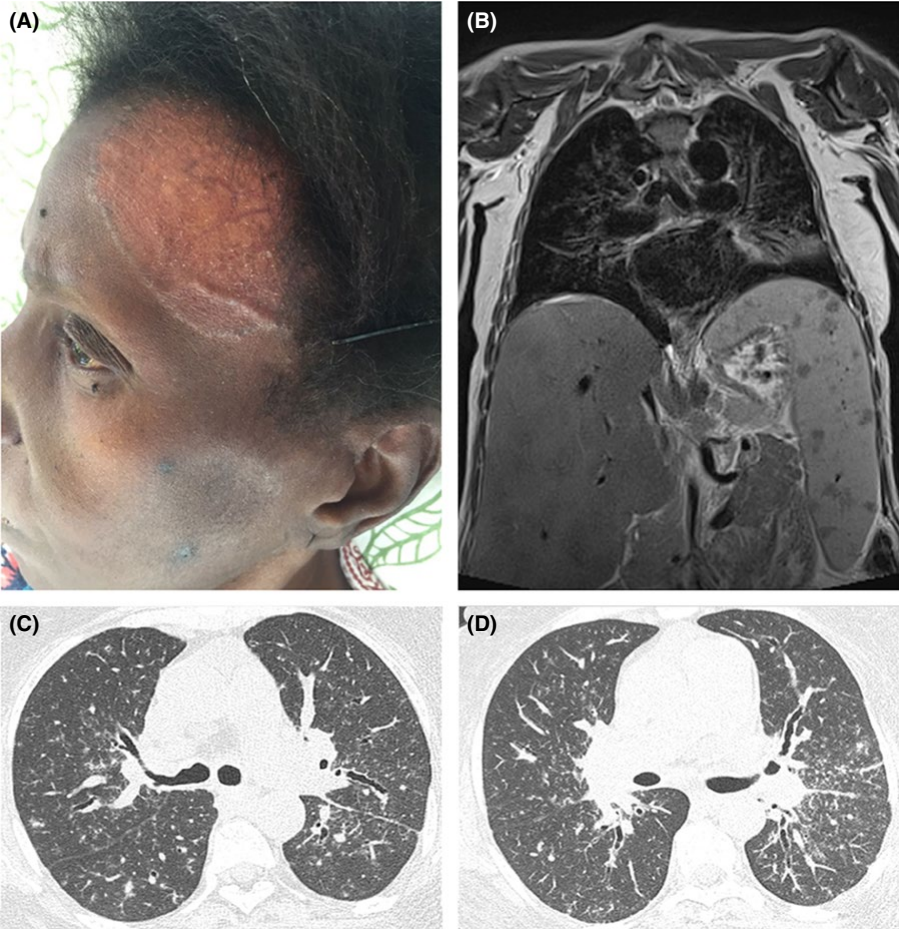
Ectoscopic examination showed a large, flat‐topped skin lesion on the forehead (A). Thoracoabdominal MRI displays hepatosplenomegaly with multiple splenic nodules, hypointense on a coronal T1‐weighted fat‐suppressed image (B). Peribronchovascular interstitial thickening was also identified. Chest CT demonstrates multiple small nodules predominating along the bronchovascular bundles (C, D). Bilateral hilar enlargement was also detected

A 48‐year‐old female patient sought medical attention due to increased abdominal size, daily fever, significant weight loss, and cholestatic jaundice initiated about 2 years previously. The patient also reported dyspnea under moderate stress. Physical examination showed hepatomegaly and erythematous plaques on the face (Figure 1A) and left inferior limb. Laboratory evaluation demonstrated elevated hepatic enzymes, hyperbilirubinemia, anemia, and polyclonal hypergammaglobulinemia. Abdominal magnetic resonance imaging (MRI) revealed hepatosplenomegaly with hypointense nodules, as well as lymph node enlargement at the splenic hilum (Figure 1B). Chest computed tomography demonstrated pulmonary nodules predominating along the bronchovascular bundles, with bilateral hilar enlargement (Figure 1C,D). Histologic samples from hepatic and cutaneous biopsies exhibited granulomas with epithelioid cells and multinucleated giant cells. The final diagnosis was sarcoidosis.

Sarcoidosis is an immune‐mediated systemic inflammatory disease of unknown etiology, characterized by noncaseating epithelioid‐cell granulomas. Sarcoidosis may affect virtually any organ system, although 90% of patients present with pulmonary involvement.^1^, ^2^, ^3^

Extrapulmonary disease is reported in 30% of patients, with the liver and spleen being the most frequently affected abdominal organs. Homogeneous hepatomegaly often associated with splenomegaly and enlarged lymph nodes is the typical imaging feature of abdominal sarcoidosis.^1^, ^3^ Multiple nodules may also be found. Cutaneous lesions occur in about 20%‐30% of patients^1^ and may assume numerous morphologic presentations. In conclusion, the clinical course of sarcoidosis is highly variable. Thus, knowledge of the clinical and radiologic features of the disease is imperative for its diagnosis and management.

## AUTHORSHIP

All the authors made substantial contribution to the preparation of this manuscript and approved the final version for submission. YMR: contributed to write the case and identify the images. GZ: performed literature search and helped in identifying appropriate images. EM: reviewed and edited the case report and helped in identifying appropriate images.

## CONSENT CONFIRMATION

Consent was obtained from the patient for publication of case details.

## CONFLICT OF INTEREST

The authors declare that they have no conflict of interests to express.
